# Supplementary material for: Dissecting the immune suppressive human prostate tumor microenvironment via integrated single-cell and spatial transcriptomic analyses
Source: Nat Commun. 2023 Feb 7;14:663. doi: 10.1038/s41467-023-36325-2 (PMC9905093; doi:10.1038/s41467-023-36325-2)
Supplement: Supplementary file 1 — Supplementary Information [file 41467_2023_36325_MOESM1_ESM.pdf]

Supplementary Information

Dissecting the immune suppressive human prostate tumor microenvironment via integrated single-cell and spatial transcriptomic analyses

Taghreed Hirz [1,2,3, §, *], Shenglin Mei [1,4, §,*] Hirak Sarkar [4], Youmna Kfoury [1,2,3], Shulin Wu [5], Bronte M. Verhoeven [6], Alexander O. Subtelny [5], Dimitar V. Zlatev [7], Matthew W. Wszolek [7], Keyan Salari [7,8], Evan Murray [8], Fei Chen [8], Evan Z. Macosko [8,9], Chin-Lee Wu [5], David T. Scadden [1,2,3], Douglas M. Dahl [7], Ninib Baryawno [6, §], Philip J. Saylor [10, §], Peter V. Kharchenko [4,2,8,11, §], David B. Sykes [1,2,3, *, §].

[§] Equally contributed authors

[*] Corresponding authors: Taghreed Hirz: THIRZ@mgh.harvard.edu; Shenglin Mei: smei8@mgh.harvard.edu; David B. Sykes: DBSYKES@mgh.harvard.edu

[1] Center for Regenerative Medicine, Massachusetts General Hospital, Boston, MA, USA

[2] Harvard Stem Cell Institute, Cambridge, MA, USA

[3] Department of Stem Cell and Regenerative Biology, Harvard University, Cambridge, MA, USA

[4] Department of Biomedical Informatics, Harvard Medical School, Boston, MA, USA

[5] Department of Pathology, Massachusetts General Hospital, Harvard Medical School, Boston, MA, USA

[6] Childhood Cancer Research Unit, Karolinska University Hospital, Stockholm, Sweden

[7] Department of Urology, Massachusetts General Hospital, Harvard Medical School, Boston, MA, USA

[8] Broad Institute of Harvard and MIT, Cambridge, MA, USA

[9] Department of Psychiatry, Massachusetts General Hospital, Boston, MA, USA

[10] Massachusetts General Hospital Cancer Center, Harvard Medical School, Boston, MA, USA

[11]Present address: Altos Labs, San Diego, CA, USA.

This file includes:
Supplementary Figures 1 to 7 and
Supplementary Table (1 to 7) Legends
Supplementary Note

Supplementary Figure 1

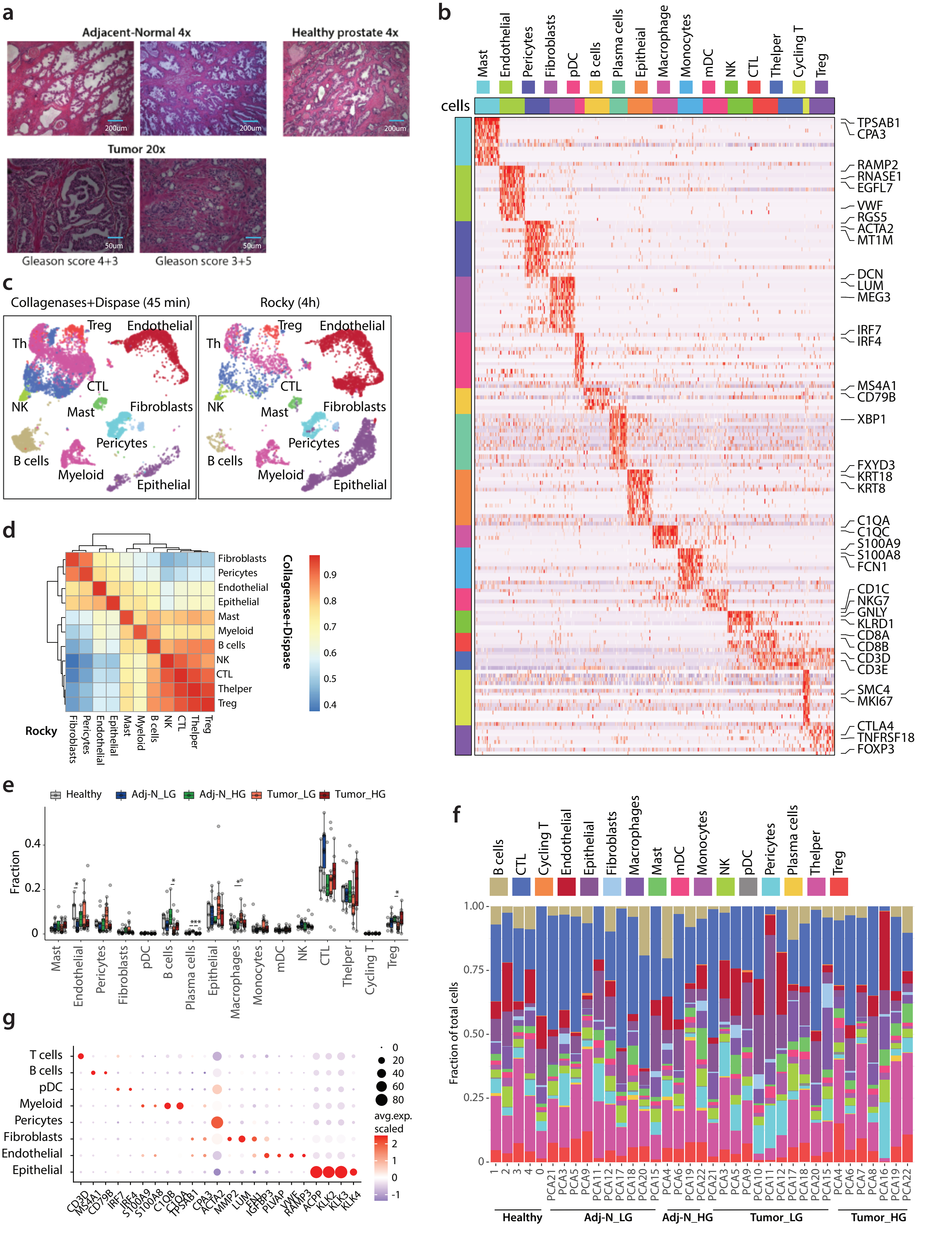

Figure S1. The prostate TME characterized by single-cell and spatial transcriptomic analyses.

a Representative photomicrographs of matched adjacent-normal prostatic tissue (A1 and B1, 4x) and prostate cancer (PCa) (A2: Gleason score 4+3 and B2: Gleason score 3+5, 20x) from radical prostatectomy specimens of two PCa patients and healthy prostate tissue from cystoprostatectomy specimen of a bladder cancer patient (C1, 4x). **b** Heatmap shows an overview of marker genes (row) expressed in major cell populations (column). **c** UMAP embedding showing major cell populations obtained using two different dissociation protocols on an adj-normal prostate tissue (Gl 3+3) separately (left: Collagenases+Dispase, right: Rocky; details in 'Methods'). **d** The Heatmap demonstrating spearman correlation coefficients of gene average expression level between Rocky (x-axis) and Collagenase+Dispase (y-axis) in each cell type. **e** Barplot representing the fraction of major cell populations within each sample fraction collected for 10x (healthy, Adj-N and tumor tissues collected from LG cases, Adj-N and tumor tissues collected from HG cases). **f** Barplot representing the fraction of major cell populations within each sample collected from 10x Genomics data. Statistical significance was accessed using two-sided Wilcoxon rank sum test (Endothelial cells **p*=0.01; Macrophage **p*=0.02; Treg **p*=0.04). **g** Dotplot representing key-marker gene expression in major cell types in Slide-seqV2. The color represents scaled average expression of marker genes in each cell type, and the size indicates the proportion of cells expressing marker genes. Source data are provided as a Source Data file.

Supplementary Figure 2

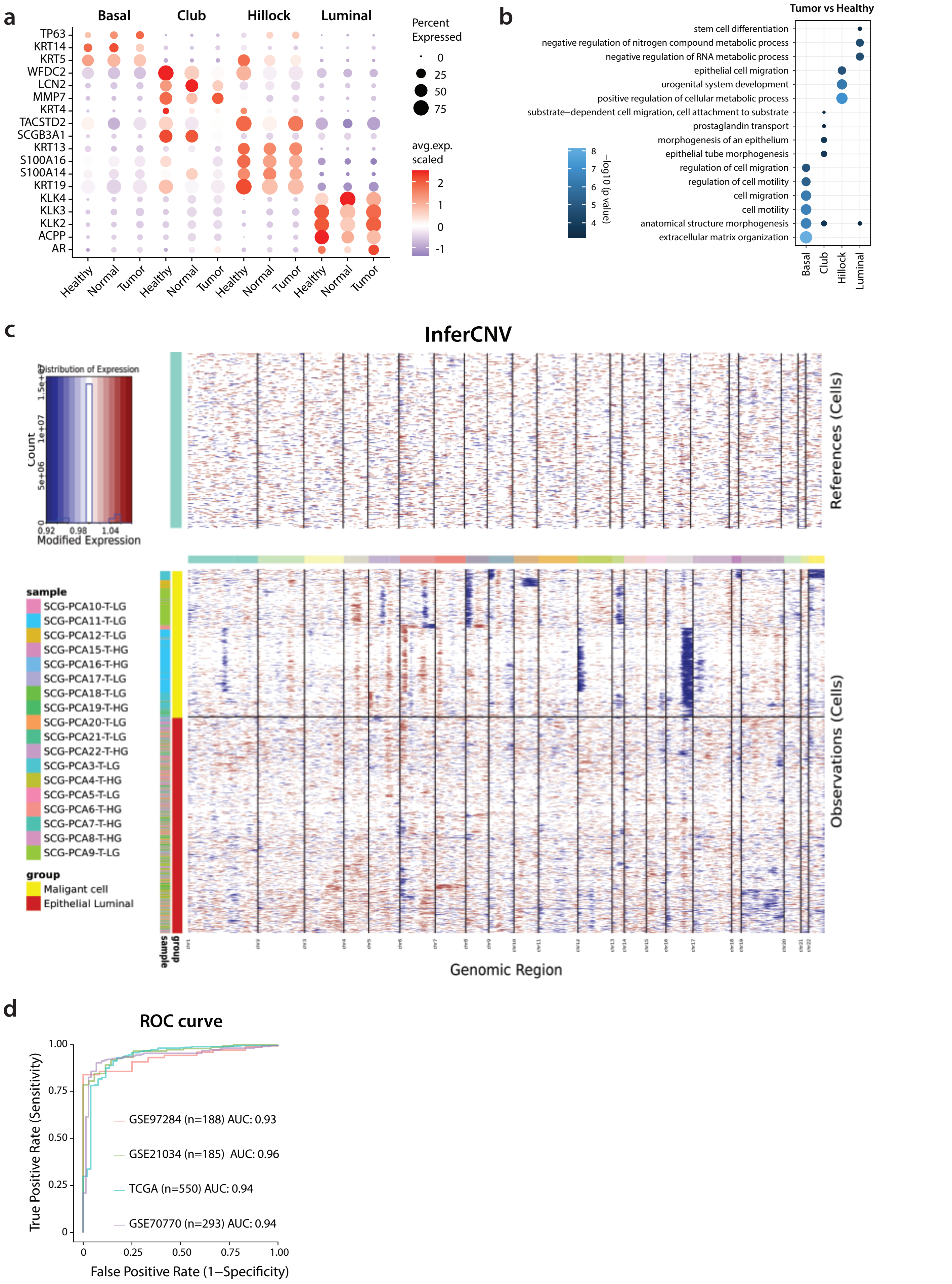

Figure S2. A Prostate Tumor Gene Signature distinguishes normal and malignant luminal epithelial cells.

a Dotplot showing the average expression of select marker genes in epithelial subpopulation across healthy, adj-normal and tumor samples. The color represents scaled average expression of select marker genes in each epithelial subpopulation, and the size indicates the proportion of cells expressing select marker genes. **b** Gene Ontology terms enriched in the top 200 high-loading differential expressed genes of the different epithelial subpopulation comparing tumor to healthy samples. **c** Inferred CNV profile of malignant cells and normal epithelial luminal cells from tumor samples, using epithelial luminal cells from healthy samples as the reference. **d** ROC curves for “Prostate Tumor Gene Signature” applied on four independent prostate cancer datasets (TCGA, GSE21034, GSE97284, GSE70770) (see 'Methods'). Source data are provided as a Source Data file.

Supplementary Figure 3

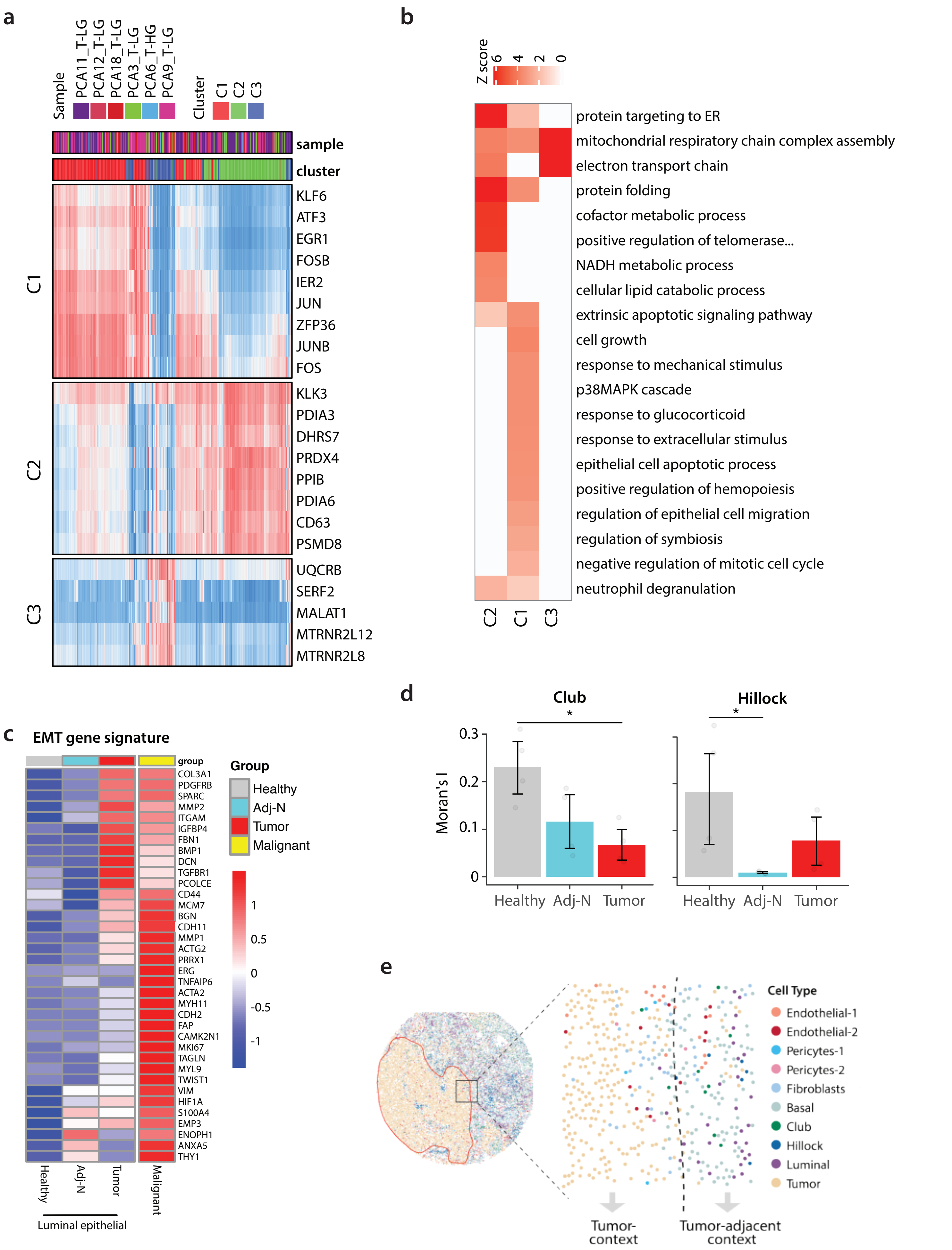

Figure S3. Heterogeneity of malignant cells.

a Heatmap showing DE genes in the three malignant cell clusters. **b** Overview of enriched GO terms of top 200 upregulated genes for each malignant cell cluster compared to all malignant cells. **c** Heatmap showing the average gene expression of EMT gene signature in malignant cells and epithelial luminal cells in healthy, adj-normal and tumor prostate samples. **d** Comparison of spatial autocorrelation (Moran's I) of epithelial club and epithelial Hillock in healthy (n=4), adj-normal (n=4) and tumor samples (n=4). Statistical analysis was accessed using two-sided Wilcoxon rank sum test (Club: *p=0.03; Hillock *p=0.03, error bars: SEM). **e** Zoomed in view of region within the tumor (HG) puck, shows the high constellation of tumor cells in the tumor-enriched region contrasting heterogeneous cell-type population on the other side. Source data are provided as a Source Data file.

Supplementary Figure 4

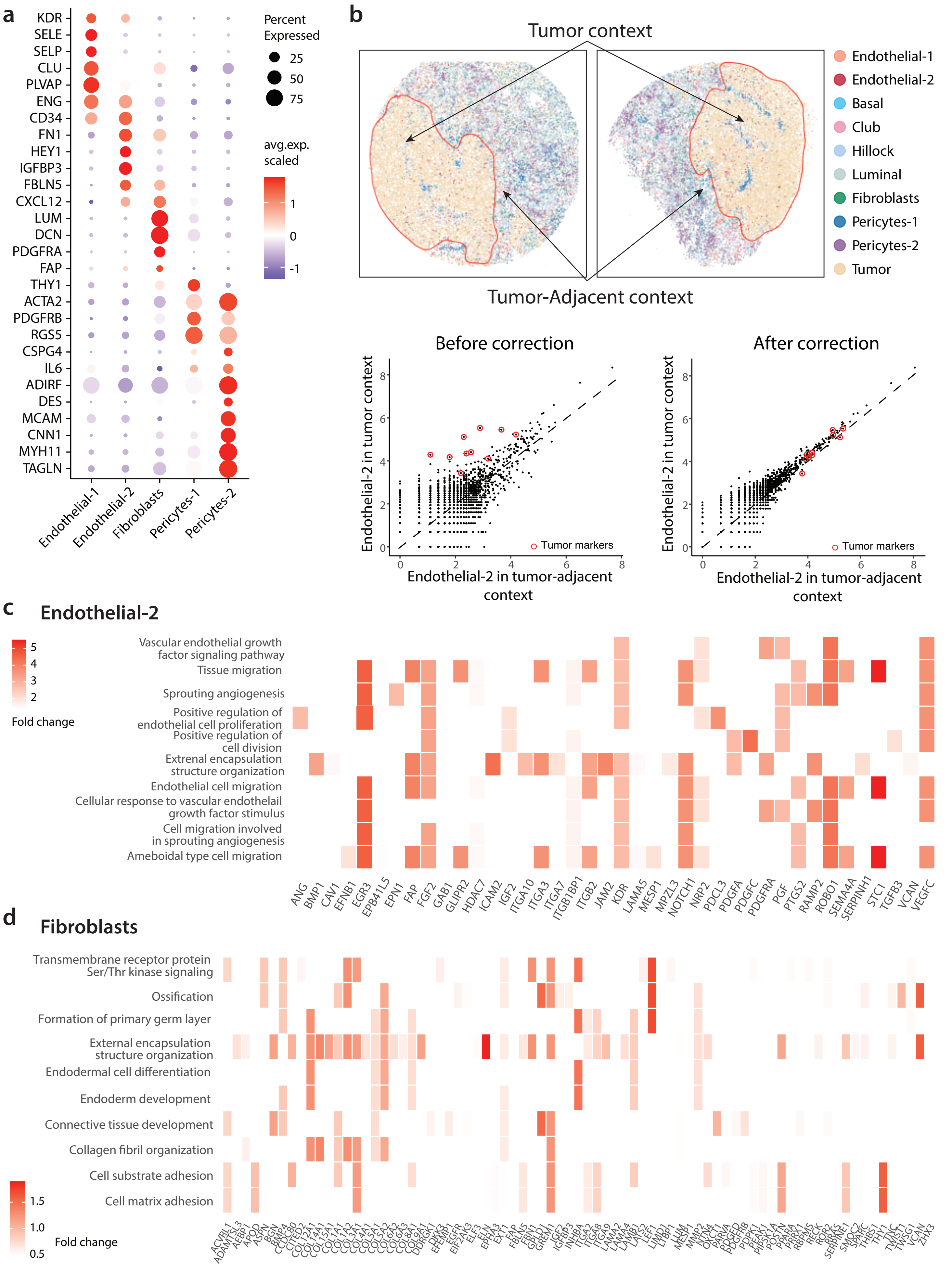

Figure S4. The prostate tumor microenvironment exhibits high endothelial angiogenic activity.

a Dotplot representing key marker gene expression in five different non-immune stromal subpopulations. The color represents scaled average expression of marker genes in each subpopulation, and the size indicates the proportion of cells expressing marker genes. **b** The differential expression tests are performed on two tumor replicates (two pucks corresponding to Tumor01 and Tumor02 from the same sample of Tumor (HG)). We model the changes in gene expression comparing the tumor context and the tumor-adjacent context. The scatterplots are showing the gene expression of Endothelial-2 cells (log-transformed pseudo-bulk) in tumor-adjacent context (x-axis) vs. the tumor-context (y-axis). The left scatter plot is with the gene expression values before correction and shows the tumor marker genes (in red) to be significantly differentially expressed while the right scatter plot is with the corrected gene expression values. **c-d** Heatmaps corresponding to upregulated genes in Endothelial-2 cells (c) and fibroblasts (d) comparing the cell location in tumor-context to tumor-adjacent context. The x-axis denotes the genes and y-axis denotes the gene-ontology pathways. The color intensity signifies the fold-change of the DE gene. Source data are provided as a Source Data file.

Supplementary Figure 5

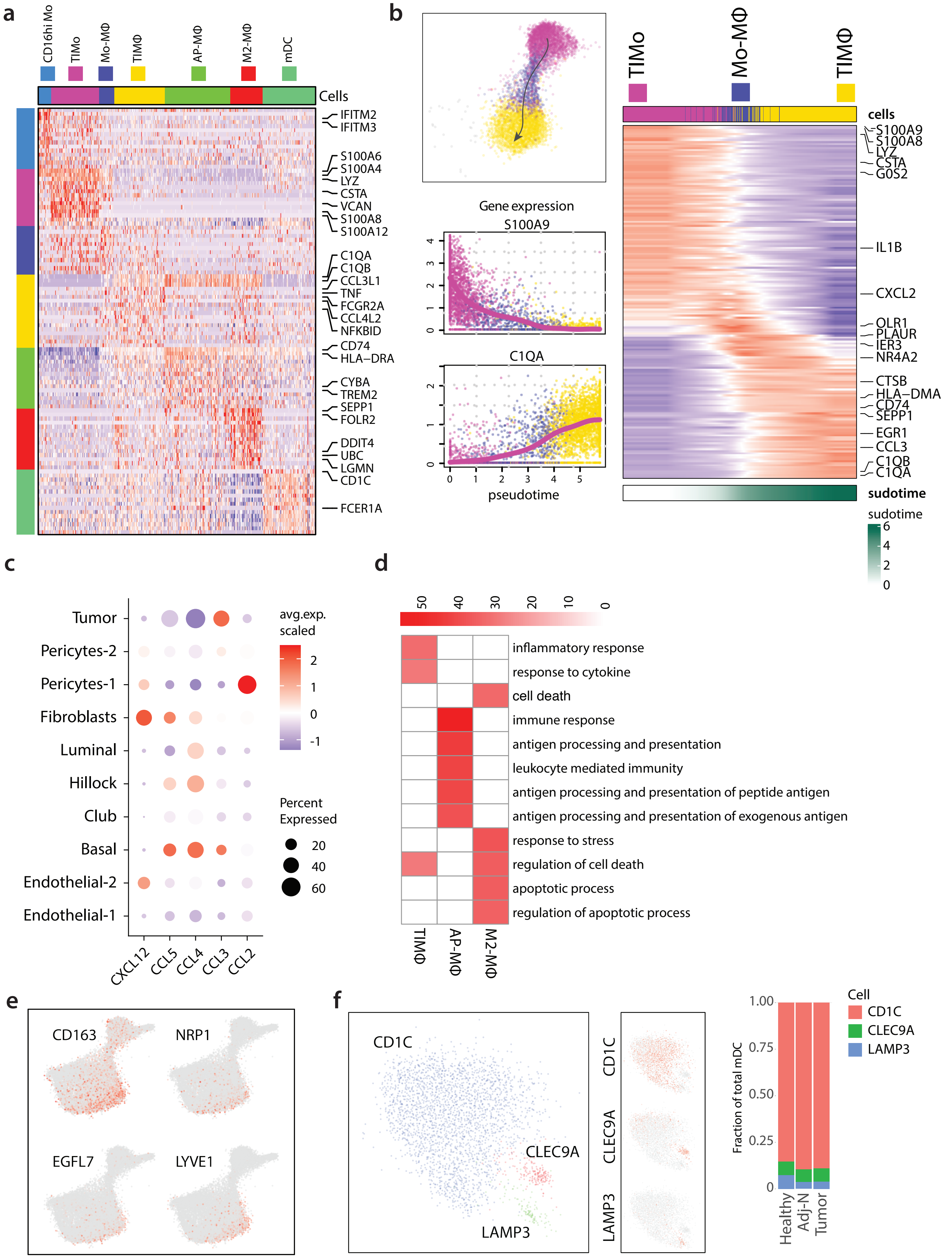

Figure S5. Immunosuppressive myeloid cells are enriched in prostate tumors.

a Heatmap shows an overview of genes (rows) differentially expressed across the myeloid subpopulations of different patients. **b** Heatmap showing the gene expression dynamics with pseudo-time moving from TIMo to TIMΦ. Representative genes are shown for each cellular state along the cell differentiation (right). Trajectory analysis demonstrates S100A9 (top) and C1QA (bottom) genes expression across pseudotime (left). **c** Dotplot showing the average expression of the indicated chemokines in the epithelial and stromal subpopulations in our dataset. The color represents scaled average expression of select marker genes in each epithelial subpopulation, and the size indicates the proportion of cells expressing select marker genes. **d** Enriched Gene Ontology BP categories for the top 200 upregulated genes in each macrophage subpopulations compared to all macrophages. **e** UMAP embedding for myeloid subpopulations showing the expression of indicated genes. **f** Detailed annotation of myeloid dendritic cells (mDCs) (left) with marker genes expression shown on UMAP embedding (middle). Right: Stacked bar plot represents the cell proportion of mDCs subpopulations across the three sample fractions. Source data are provided as a Source Data file.

Supplementary Figure 6

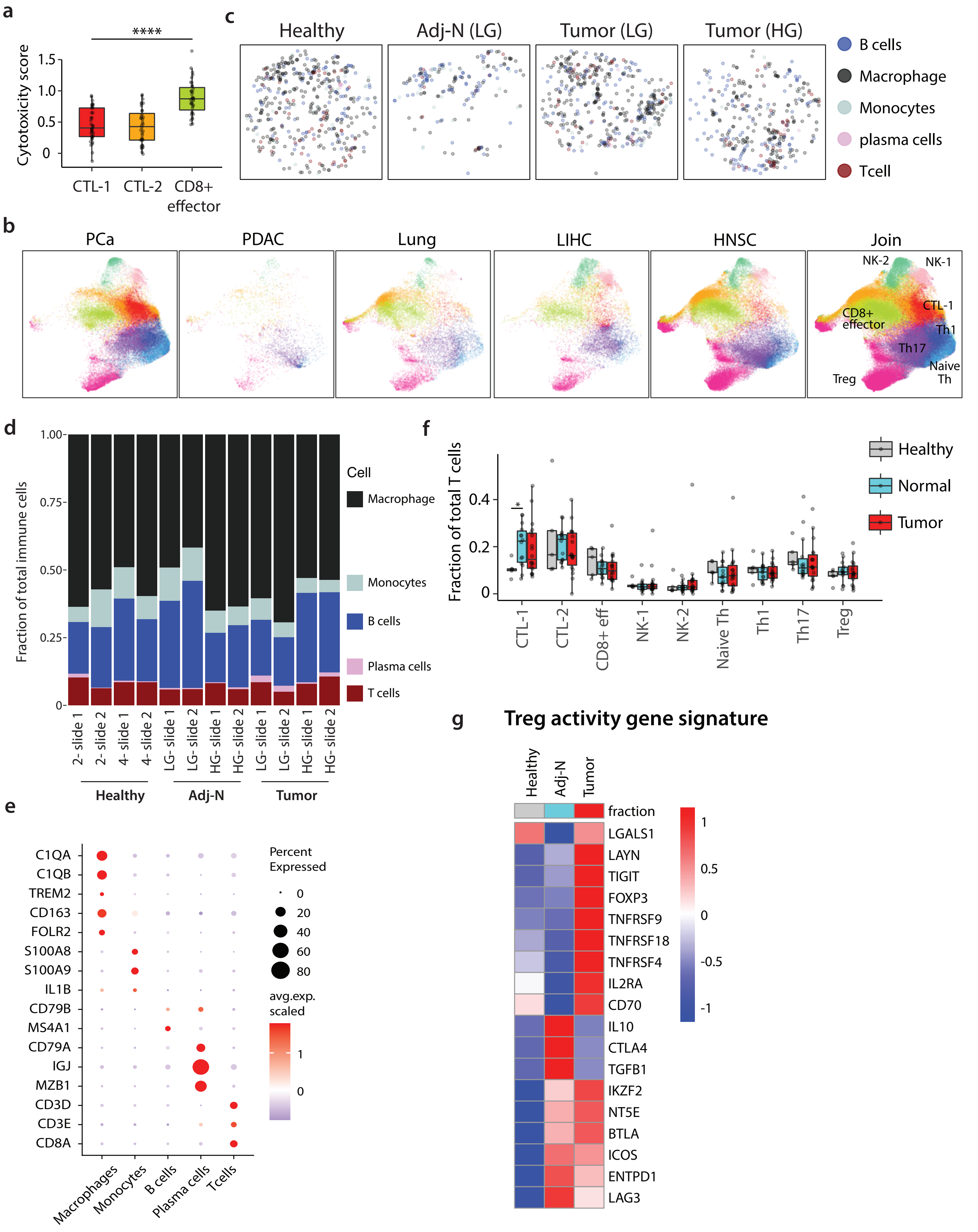

Figure S6. Prostate cancer is characterized by T-cell exhaustion and immunosuppressive Treg activity.

a Boxplots represent the average expression of cytotoxicity score in CD8+ subpopulations (CTL-1 n=37, CTL-2 n=36 and CD8+ effector cells n=35). Boxplots include centerline, median; box limits, upper and lower quartiles; and whiskers are highest and lowest values no greater than 1.5x interquartile range. Statistical significance was accessed using two-sided Wilcoxon rank sum test (CTL-1 vs CD8+ effector ****p=4.08E-09) **b** Joint embedding showing the detailed annotation of lymphoid subpopulations in different cancer types including prostate cancer (PCa), pancreatic ductal adenocarcinoma (PDAC), Head and Neck squamous cell carcinoma (HNSCC), liver hepatocelluar carcinoma (LIHC), lung cancer (lung) and join (all samples). **c** Spatial presentation at a high-resolution level using Slide-seqV2 for immune populations in healthy, adj-normal and two tumor tissues collected from a low-grade (Tumor-LG) and high-grade (Tumor-HG) patients. Patinets ID from Supplementary Data 2 represented here as healthy is HP1, adj-normal of LG case is Benign04, tumor tissue of LG case is Tumor08, tumor tissue of HG case is Tumor02. **d** Stacked bar plot represents the changes in the cell proportion of immune populations obtained from Slide-seqV2 data across the healthy, adj-normal and tumor samples collected from low and high-risk patients. **e** Dot plot representing key marker gene expression in immune subpopulations in Slide-seqV2 data. The color represents scaled average expression of marker genes in each cell type, and the size indicates the proportion of cells expressing marker genes. **f** Boxplot comparing the relative abundance of different lymphoid subpopulations across healthy (n=5), adj-normal (n=14) and tumor (n=18) samples. Boxplots include centerline, median; box limits, upper and lower quartiles; and whiskers are highest and lowest values no greater than 1.5x interquartile range. Statistical significance was accessed using two-sided Wilcoxon rank sum test (CTL-1 *p=0.03). G. Heatmap shows average expression of “Treg activity gene signature” (row) in Treg subpopulation in the three different samples (column). Source data are provided as a Source Data file.

Supplementary Figure 7

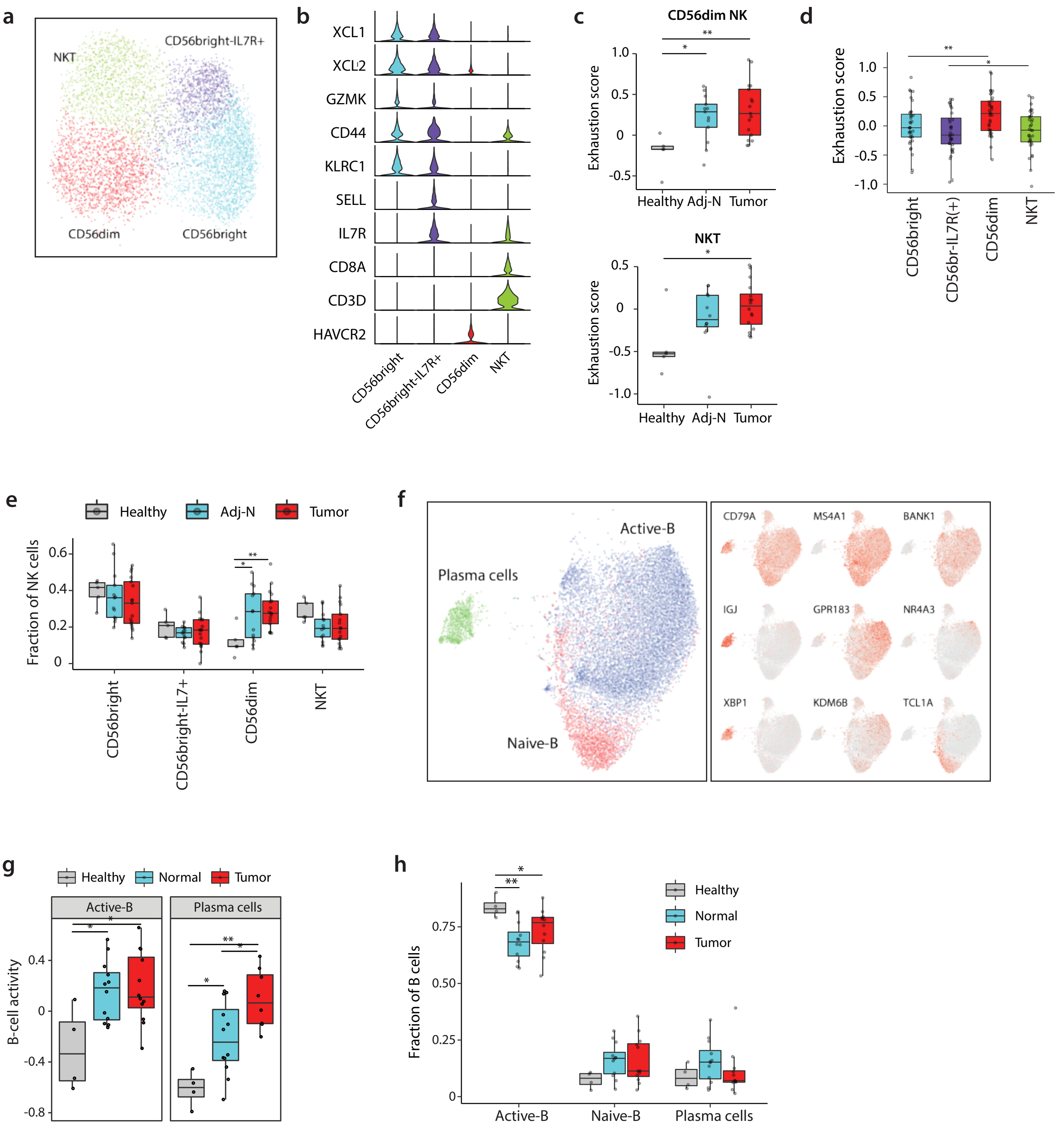

Figure S7. The prostate cancer TME is enriched by exhausted CD56DIM NK cells and activated B cells.

a Joint embedding showing the detailed annotation of NK subpopulations. **b** Violin plot showing the average expression of indicated marker genes in NK subpopulations. **c** Boxplots comparing the exhaustion score of CD56dim and NKT subpopulation across healthy (n=5), adj-normal (n=14), and tumor (n=18) samples. See Supplementary Data 4 for the genes defining exhaustion score. Statistical significance was accessed using two-sided Wilcoxon rank sum test (CD56Dim NK *p=0.02, **p=0.001; NKT *p=0.02). **d**. Boxplot comparing the average expression of the exhaustion score between the different NK subpopulations. Statistical significance was accessed using two-sided Wilcoxon rank sum test (CD56bright vs CD56dim CD16+ **p=0.009; CD56bright-IL7R+ vs CD56dim CD16- *p=0.03). **e** Boxplot comparing the relative abundance of different NK subpopulations in healthy (n=5), adj-normal (n=14), and tumor (n=18) samples. Statistical significance was accessed using two-sided Wilcoxon rank sum test (CD56dim: *p=0.04, **p=0.002). **f** Joint embedding showing the detailed annotation of B cell subpopulations (left) and the expression of B cell specific marker genes (right). **g** Boxplot comparing B cell activity signature in active B and plasma subpopulations between healthy (n=5), adj-normal (n=14), and tumor (n=18) samples. Statistical significance was accessed using two-sided Wilcoxon rank sum test, p-values could be found at Supplementary Data 6. **h** Boxplot comparing the relative abundance of each B subpopulations across healthy (n=5), adj-normal (n=14) and tumor (n=18) samples. Statistical significance was accessed using two-sided Wilcoxon rank sum test (Active-B: *p=0.03, **p=0.004). Boxplots in (**c-e, g-h**) include centerline, median; box limits, upper and lower quartiles; and whiskers are highest and lowest values no greater than 1.5x interquartile range. Source data are provided as a Source Data file.

Description of Additional Supplementary Files

Title: Supplementary Data 1.

Description: Clinical characteristics of healthy donors and prostate cancer patients.

Title: Supplementary Data 2.

Description: Sequencing information and data quality control of individual samples.

Title: Supplementary Data 3.

Description: Gene list used for cell annotations.

Title: Supplementary Data 4.

Description: List of gene sets for gene signature score.

Title: Supplementary Data 5.

Description: Predicted ligand and receptor interaction channels.

Title: Supplementary Data 6.

Description: Table of statistical significances.

Title: Supplementary Data 7.

Description: Antibodies used for cell sorting

Supplementary Note: Context-dependent differential expression with linear admixture correction

Hirz *et al.*

Abstract

Slide-Seq [1] data captures transcriptional profiles of tissue sections in a spatially-resolved manner. A key advantage of such approach is the ability to examine how the expression state of different cell types depends on their context. For instance, the state of an immune cell may differ depending on whether it is found in local inflammatory or non-inflammatory environment. Here we describe an approach for carrying out such tests based on Slide-seqV2 platform.

Introduction

Slide-seqV2 relies on mRNA hybridization to barcoded bead arrays. The beads are packed on a surface of a glass slide in a circular pattern, forming a "puck". The beads are approximately $10\mu m$ in size and are packed so densely that the distance between bead centers is comparable. Despite such high spatial resolution of the features, inferring transcriptional state of individual cells is challenging due to the fact that multiple cells may contribute to an individual barcoded bead. Figure 1 demonstrates the challenge. A cell may physically overlap more than one bead, and lateral mRNA diffusion during hybridization can further spread mRNA from a given cell to nearby beads. As a result, transcriptional profiles assessed on an individual bead are likely to report a mixture of material from different cells.

A number of computational methods have been developed recently to identify "pure" beads - those with material coming predominantly from a single cell type[2,3]. Such "deconvolution" methods can provide good certainty in the identity of the dominant cell type, however, cannot necessarily discern the detailed transcriptional state of a cell from admixed profiles. Such detailed transcriptional features are central to analysis of how the state of a cell is influenced by its tissue context. For example, consider the state of fibroblast cells in two different contexts (Figure 1a): Tumor context, dominated in its composition by the presence of tumor cells, and Tumor-adjacent context which in addition to fibroblasts contains epithelial and endothelial cells. Even for the beads predominantly capturing material from fibroblast cells (red bead, 1b), the transcriptional profiles captured by the beads will systematically differ between the two context, with the fibroblast beads in the Tumor context capturing substantial signal from adjacent tumor cells, while firboblast beads in the Tumor-adjacent context will capture admixture from epithelial and endothelial neighbors.

To accurately evaluate the transcriptional difference of a given cell type between two contexts, one must correct for the systematic differences in the admixtures. To do so, we use a linear mixture model to estimate coefficients of composition of different cell-types present in the two contexts and correct for them. To simplify the computational problem, we assume that the mixture from the cells to their nearby bead happens in a linear manner, therefore the measured gene expression of each bead is a linear mixture of the nearby cells. Instead of approaching the problem at a single-bead level, we consider average profiles of all beads of a given type in a given context (e.g. by forming pseudo-bulk profiles for all "fibroblast" beads in Tumor context). This is sufficient for answering questions about average differential expression between contexts, and carries two notable advantages. First, pseudo-bulk formulation reduces uncertainty and computational burden. More importantly, together with a linear admixture assumption mentioned above, such formulation allows to avoid the issue of estimating pure admixture profiles. For instance, if endothelial cells exist in both contexts, they may contribute to the target firboblast beads in both contexts and hence would need to be taken into account when correcting the difference. However, the true expression profile of the endothelial cells is also unknown and challenging to estimate, as even "pure" endothelial beads will carry admixture from other cell types, including for example, tumor cells. This sets up a circular dependency that would be difficult to resolve. However, as we will show, under a linear admixture assumption, all such secondary admixtures will cancel out.

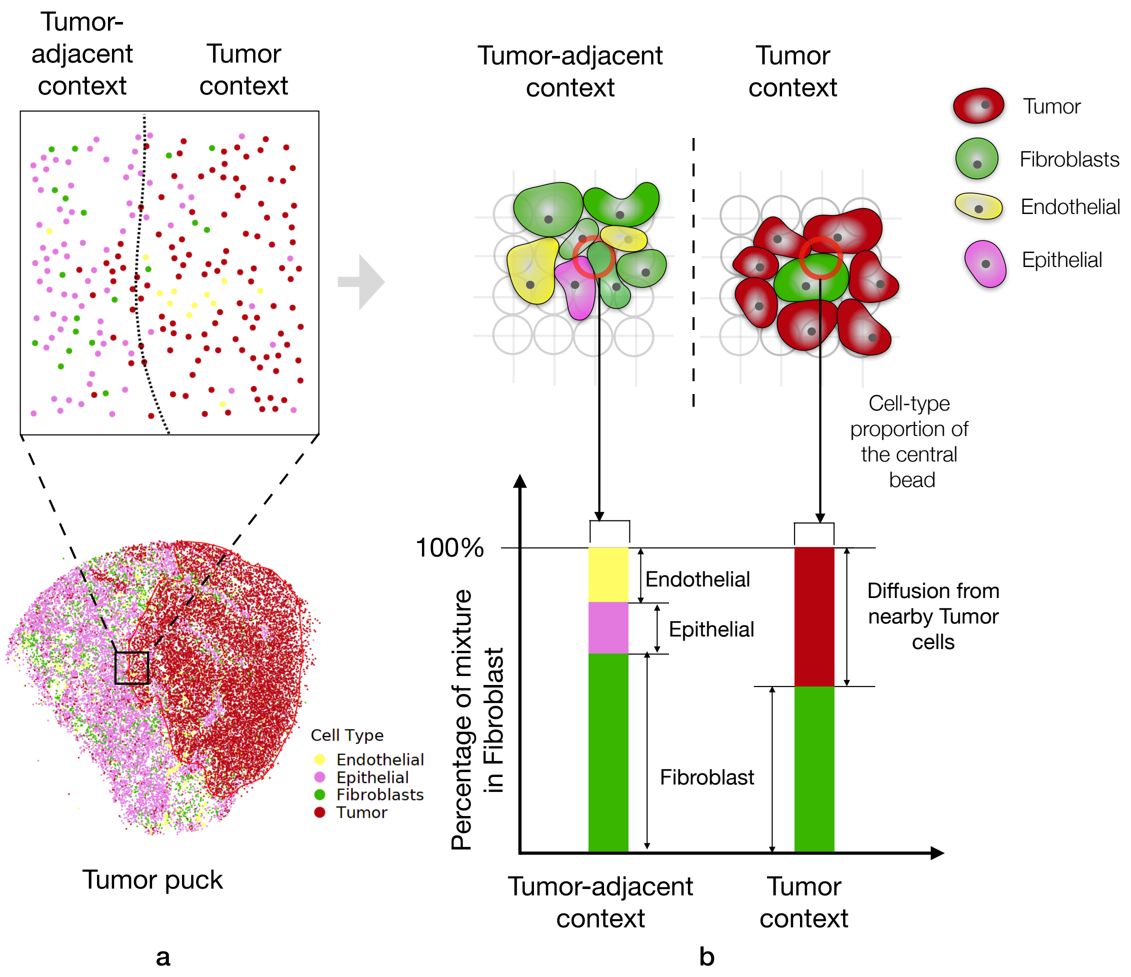

Figure 1. Segmenting target cell-type (Tumor) by fitting a kernel density estimator around the spatial locations of the target cell type.

Computational Pipeline

Annotation and Segmentation

The initial annotation of the Slide-seq beads is carried out by an existing tool RCTD[2], based on annotated scRNA-seq data. Given an annotated spatial dataset, a spatial "context" is defined depending on the organization of the tissue and the biological question at hand. In the current manuscript we are interested in investigating the effect of Tumor micro-environment (TME) on other cell-types. We, therefore, defined the contexts on the basis of the localization of the Tumor cells. As Figure 2 shows, the segmented region (within red boundary) is defined to be the "tumor context", where as the region outside that boundary is defined as as "tumor-adjacent context". Please note that depending on the biological question one sets out to ask, the definition of context and the exact way of determining the segments will change. Contexts may also be spread out, based on local neighborhood properties instead of large-scale segments.

In order to set up the context-dependent differential expression problem, we require three inputs:

1. Gene expression profiles of different cells, formally defined as a matrix M of integer values, of dimension $m \times n$ where there are m cells and n genes
2. Corresponding cell-type annotation for each cell denoted by a set $\mathscr{C}$
3. A set of contexts D, defined as a map from the cell id to a discrete variable denoting the context. For example, Figure 2a shows the annotated Slide-seqV2 experiment from the main manuscript, consisting of 10 cell-types, and 2 contexts, namely tumor-adjacent context and tumor context.

Given such a formalization, we first create a composite class for each cell, combining the cell-type of a cell and the context assigned to the cell. As each cell-type can be potentially present in any context, the combined classification will create $|\mathscr{C}||D|$ categories. Next we create pseudo-bulk profiles by summing up (for each gene) the molecules detected in all of the cells within one of the $|\mathscr{C}||D|$ categories. This operation would produce the matrix M' of dimension $n \times |\mathscr{C}||D|$. Note that each column vector of this matrix denotes the pseudo-bulk expression of the cells for a particular cell-type under a specific context. As the column vector of this matrix is of special interest to us, we define the column vector of M' corresponding to cell-type c_i and context d_j as κ_{c_i,d_j}.

Given a set of contexts and a particular "target" cell-type, we set up a linear model specific to the "target" cell-type. Using the same notations, given 2 contexts $\{d_1, d_2\}$, we first consider a subset of the columns of M', to only include the cell-types within these contexts. Let's denote the reduced matrix as M'_{d_1,d_2} of dimension $n \times 2|\mathscr{C}|$.

Regression based correction

To find out the differentially expressed genes for the target cell-type c_i in context d_1, when compared to d_2, we calculate the following quantities: a matrix L of dimension $n \times (2|\mathscr{C}|-1)$ with all the columns of M'_{d_1,d_2} except the one that corresponds to the cell-type c_i and the context d_1; a vector κ_{c_i,d_1} of length n, containing the gene expression for the cell-type c_i in the context d_1. Using L and κ_{c_i,d_1} we then seek a vector η of length $2|\mathscr{C}|-1$ by solving the following constrained optimization problem:

$$\min_{\eta} \|L\eta - \kappa_{c_i,d_1}\| \quad \text{s.t.} \begin{cases} -\infty \leq \eta_j \leq 0 & \text{if } j \in d_2 \text{ and } j \notin c_i \\ 0 \leq \eta_j \leq \infty & \text{Otherwise} \end{cases} \tag{1}$$

The optimization described in the equation 1 can be solved by a bounded-value least square optimization, which we perform by using the `bvls`[4] package in R. The estimated η is then used to correct the pseudo bulk profile of the target cell-type c_i, in the contexts d_1 and d_2 by constructing the regressed profiles $\hat{\kappa}_{c_i,d_1}$ and $\hat{\kappa}_{c_i,d_2}$, respectively. Specifically, we compute two vectors η^+ and η^- of length $|\eta|$ as follows:

$$\eta_j^+ = \begin{cases} \eta_j & \text{if } \eta_j > 0 \\ 0 & \text{otherwise} \end{cases} \tag{2}$$

and

$$\eta_j^- = \begin{cases} \eta_j & \text{if } \eta_j < 0 \\ 0 & \text{otherwise} \end{cases} \tag{3}$$

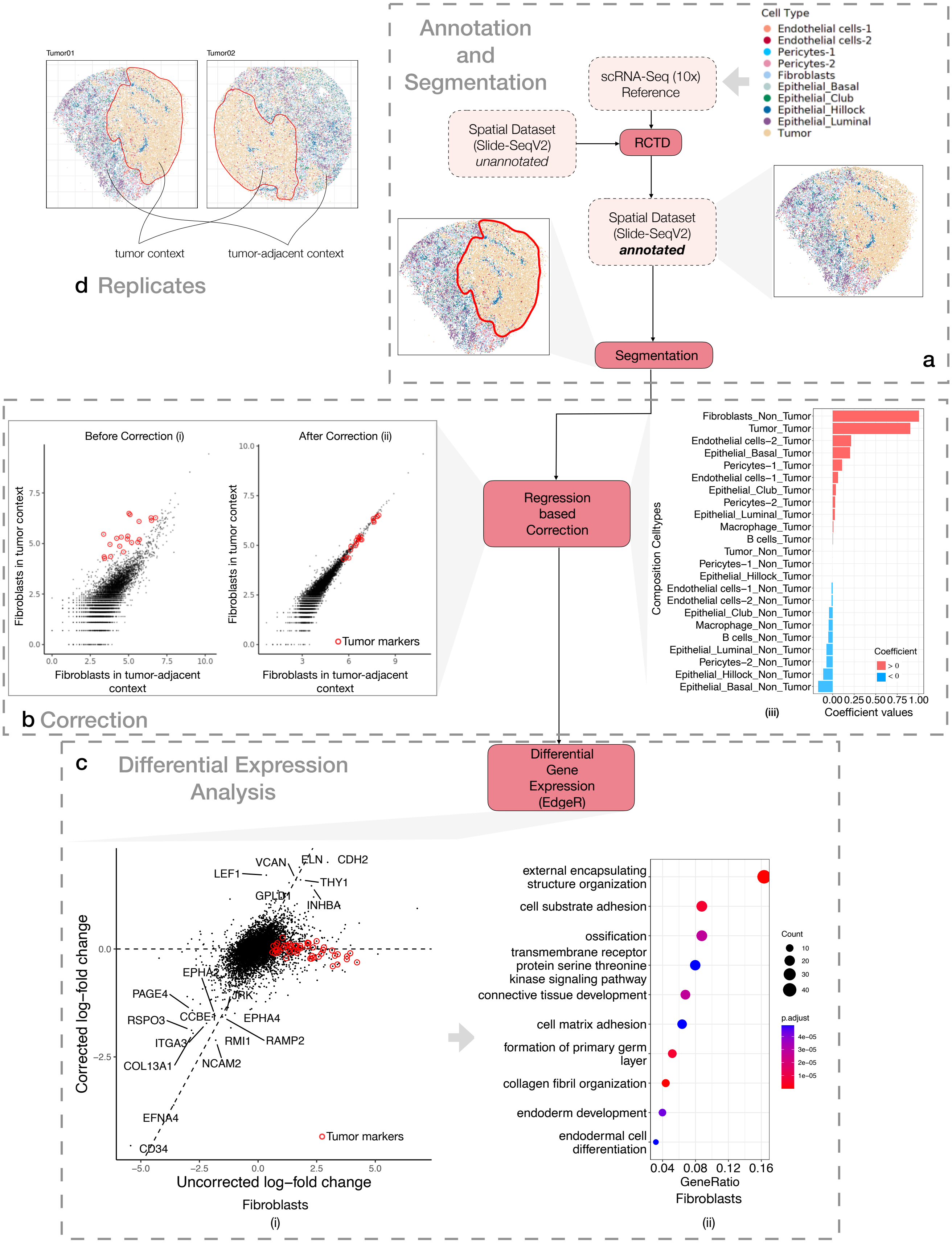

Figure 2. The overview of the Slide-seqV2 based computational pipeline.

The $\hat{\kappa}_{c_i,d_1}$ and $\hat{\kappa}_{c_i,d_2}$ are computed

$$\hat{\kappa}_{c_i,d_1} = \kappa_{c_i,d_1} - \eta^- L$$
$$\hat{\kappa}_{c_i,d_2} = \eta^+ L$$

The plots in Figure 2b show regression-based correction for the prostate cancer datasets from the main manuscript Specifically, we look for expression differences in the fibroblast target cell type, between the tumor context and the tumor-adjacent contexts. The barplot in Figure 2b(iii) shows the coefficients η for the cell types in each of the two contexts. Figure 2b shows two scatter plots. Each point corresponds to a gene. The left scatter plot shows expression of each gene in $\kappa_{\text{Fibroblasts,tumor-adjacent}}$ (x-axis) vs $\kappa_{\text{Fibroblasts,tumor}}$ (y-axis). In these uncorrected pseudo-bulk profiles we see inflated expression values for tumor cell markers (denoted by red circles, e.g. TMEFF2, NPY, ERG etc.) demonstrating the phenomenon shown in Figure 1b(i). The other scatter plot (1b(ii)) shows analogous contrast between contexts using corrected gene expressions $\hat{\kappa}_{\text{Fibroblasts,tumor-adjacent}}$ vs $\hat{\kappa}_{\text{Fibroblasts,tumor}}$, where the Tumor markers are no longer showing up as being significantly different in their expression.

Differential Expression

To test for differentially expressed genes, the corrected profiles $\hat{\kappa}_{c_i,d_1}$ and $\hat{\kappa}_{c_i,d_2}$ are passed to `EdgeR`[5]. When there are more than one Slide-seq replicate present, we apply the regression-based correction for individual replicate before running the DE tool. If run without replicates, the biological variation parameter (`bcv`) within the `EdgeR` is set to 0.1.

Figure 2c shows the results obtained from `EdgeR`[5] based on the corrected Fibroblast profiles from the two HG tumor pucks (Figure 2d). We have also run `EdgeR` with the uncorrected profiles to trace the improvements achieved from the correction. The scatter plot on the left side of Figure 2c(i) shows the log-fold change computed by `EdgeR` with the uncorrected (x-axis) and the corrected profiles (y-axis). Similar to Figure 2b, the tumor cell marker genes are marked by red circles. The log-fold change for the tumor marker genes is reduced with the corrected profiles. Once computed, the top differentially expressed genes could be used for gene enrichment and other downstream analysis (Figure 2c(ii)).

References

1. Stickels, R. R. *et al.* Highly sensitive spatial transcriptomics at near-cellular resolution with slide-seqv2. *Nat. biotechnology* **39**, 313–319 (2021).
2. Cable, D. M. *et al.* Robust decomposition of cell type mixtures in spatial transcriptomics. *Nat. Biotechnol.* 1–10 (2021).
3. Dong, R. & Yuan, G.-C. Spatialdwls: accurate deconvolution of spatial transcriptomic data. *Genome biology* **22**, 1–10 (2021).
4. Stark, P. B. & Parker, R. L. Bounded-variable least-squares: an algorithm and applications. *Comput. Stat.* **10**, 129–129 (1995).
5. Robinson, M. D., McCarthy, D. J. & Smyth, G. K. edger: a bioconductor package for differential expression analysis of digital gene expression data. *Bioinformatics* **26**, 139–140 (2010).
