# Supplementary material for: Dissecting the immune suppressive human prostate tumor microenvironment via integrated single-cell and spatial transcriptomic analyses
Source: Nat Commun. 2023 Feb 7;14:663. doi: 10.1038/s41467-023-36325-2 (PMC9905093; doi:10.1038/s41467-023-36325-2)
Supplement: Supplementary file 3 — Description of Additional Supplementary Files [file 41467_2023_36325_MOESM3_ESM.pdf]

Description of Additional Supplementary Files

Title: Supplementary Data 1.

Description: Clinical characteristics of healthy donors and prostate cancer patients.

Title: Supplementary Data 2.

Description: Sequencing information and data quality control of individual samples.

Title: Supplementary Data 3.

Description: Gene list used for cell annotations.

Title: Supplementary Data 4.

Description: List of gene sets for gene signature score.

Title: Supplementary Data 5.

Description: Predicted ligand and receptor interaction channels.

Title: Supplementary Data 6.

Description: Table of dataset statistics.

Title: Supplementary Data 7.

Description: Antibodies used for cell sorting.
