# Supplementary material for: Dissecting the immune suppressive human prostate tumor microenvironment via integrated single-cell and spatial transcriptomic analyses
Source: Nat Commun. 2023 Feb 7;14:663. doi: 10.1038/s41467-023-36325-2 (PMC9905093; doi:10.1038/s41467-023-36325-2)
Supplement: Supplementary file 11 — Reporting Summary [file 41467_2023_36325_MOESM11_ESM.pdf]

nature portfolio

Corresponding author(s): Taghreed Hirz, Shenglin Mei, David B. Sykes

Last updated by author(s): Jan 12, 2023

Reporting Summary

Nature Portfolio wishes to improve the reproducibility of the work that we publish. This form provides structure for consistency and transparency in reporting. For further information on Nature Portfolio policies, see our Editorial Policies and the Editorial Policy Checklist.

Statistics

For all statistical analyses, confirm that the following items are present in the figure legend, table legend, main text, or Methods section.

n/a	Confirmed	
☐	☒	The exact sample size (n) for each experimental group/condition, given as a discrete number and unit of measurement
☐	☒	A statement on whether measurements were taken from distinct samples or whether the same sample was measured repeatedly
☐	☒	The statistical test(s) used AND whether they are one- or two-sided<br>*Only common tests should be described solely by name; describe more complex techniques in the Methods section.*
☒	☐	A description of all covariates tested
☐	☒	A description of any assumptions or corrections, such as tests of normality and adjustment for multiple comparisons
☐	☒	A full description of the statistical parameters including central tendency (e.g. means) or other basic estimates (e.g. regression coefficient) AND variation (e.g. standard deviation) or associated estimates of uncertainty (e.g. confidence intervals)
☐	☒	For null hypothesis testing, the test statistic (e.g. F, t, r) with confidence intervals, effect sizes, degrees of freedom and P value noted<br>*Give P values as exact values whenever suitable.*
☒	☐	For Bayesian analysis, information on the choice of priors and Markov chain Monte Carlo settings
☒	☐	For hierarchical and complex designs, identification of the appropriate level for tests and full reporting of outcomes
☐	☒	Estimates of effect sizes (e.g. Cohen's d, Pearson's r), indicating how they were calculated

Our web collection on statistics for biologists contains articles on many of the points above.

Software and code

Policy information about availability of computer code

Data collection	NextSeq 500 Sequencing System (Illumina) and bcl2fastq 1.8.4 were used to acquire data.
Data analysis	The sequenced 10x libraries were mapped to GRCh38 human genome using Cell Ranger software (version 3.0.1), Conos were used to integrate multiple scRNA-seq datasets. inferCNV (version 1.3.3) was used to predict copy number variations (CNVs). We perform RNA velocity analysis using python package velocity (version 0.17) and scvelo (version 0.2.3). Slide-seq data was annotated using RCTD (version 2.0.0). Statistics significance tests and plots were generated with R (version 4.1.1). Code related to this manuscript can be found at https://github.com/shenglinmei/ProstateCancerAnalysis. Spatial Differential gene expression analysis can be found at https://github.com/kharchenkolab/slideseqde. For in vivo data, statistical analysis was done using Prism v9.5.0. Flow sorting for live-nonerythroid cells (DAPI-neg/CD235-neg) was performed on a BD FACS Aria III instrument (version 8.0.3) equipped with a 100um nozzle (BD Biosciences, San Jose, CA). All flow cytometry data were analyzed using FlowJo software (FlowJo 10.8.1, Treestar, San Carlos, CA).

For manuscripts utilizing custom algorithms or software that are central to the research but not yet described in published literature, software must be made available to editors and reviewers. We strongly encourage code deposition in a community repository (e.g. GitHub). See the Nature Portfolio guidelines for submitting code & software for further information.

Data

Policy information about availability of data

All manuscripts must include a data availability statement. This statement should provide the following information, where applicable:

- Accession codes, unique identifiers, or web links for publicly available datasets
- A description of any restrictions on data availability
- For clinical datasets or third party data, please ensure that the statement adheres to our policy

Raw single-cell RNA sequencing data and processed data can be accessed from the NCBI Gene Expression Omnibus database : GSE181294 (token: stofmiyuhzwjfut). GRCh38 human reference genome was download from 10X genomics (https://support.10xgenomics.com/single-cell-gene-expression/software/downloads/). For the joint alignment analysis with public scRNAseq data. We downloaded raw count matrix for PDAC (https://ngdc.cncb.ac.cn/bioproject/browse/PRJCA001063), LIHC(https://www.ncbi.nlm.nih.gov/geo/query/acc.cgi?acc=GSE140228), LUSC(https://www.ebi.ac.uk/biostudies/arrayexpress/studies/E-MTAB-6149), HNSC(https://www.ncbi.nlm.nih.gov/geo/query/acc.cgi?acc=GSE139324). Prostate cancer bulk RNAseq and microarray data are download from the NCBI Gene Expression Omnibus database: GSE21034 (https://www.ncbi.nlm.nih.gov/geo/query/acc.cgi?acc=GSE21034), GSE97284 (https://www.ncbi.nlm.nih.gov/geo/query/acc.cgi?acc=GSE97284), TCGA (https://www.cbioportal.org/study/clinicalData?id=prad_tcga), GSE70770 (https://www.ncbi.nlm.nih.gov/geo/query/acc.cgi?acc=GSE70770).

Human research participants

Policy information about studies involving human research participants and Sex and Gender in Research.

Reporting on sex and gender	All samples in our study were collected from males.
Population characteristics	All prostate cancer patients were males at the ages of 46-73. Healthy prostate tissues were collected from cystoprostatectomy surgeries (4 samples) and one from autopsy. All patients' clinical characterization are included in Supplementary Data 1.
Recruitment	Patients clinically indicated for prostatectomy surgeries (prostate removal) or cystoprostatectomy surgeries due to bladder cancer (bladder+prostate removal) were asked if specimens could be collected and used for the intended study. The autopsy was collected from MGH rapid autopsy program. All the human specimens and organisms involved in this study were allocated in randomization.
Ethics oversight	In accordance with the U.S. Common Rule and after Institutional Review Board (IRB) approval, all human tissues were collected at Massachusetts General Hospital (MGH, Boston, MA) and carried out with institutional review board (IRB) approval (IRB#2003P000641). Written informed consents were obtained from all participants in the study.

Note that full information on the approval of the study protocol must also be provided in the manuscript.

Field-specific reporting

Please select the one below that is the best fit for your research. If you are not sure, read the appropriate sections before making your selection.

☒ Life sciences ☐ Behavioural & social sciences ☐ Ecological, evolutionary & environmental sciences

For a reference copy of the document with all sections, see nature.com/documents/nr-reporting-summary-flat.pdf

Life sciences study design

All studies must disclose on these points even when the disclosure is negative.

Sample size	39 single cell RNA-seq samples and 12 Slide-seqV2 samples were included in this study. Sample sizes were chosen in a manner commensurate with similar previous studies (Bi et al., 2021, and Chen et al., 2021).
Data exclusions	We applied two general quality measures on raw gene-cell-barcode matrix for each cell: Total UMI > 600 and Scrublet scores < 0.4. PCA24 (PCA24-N-LG_Collagenases+Dispase, PCA24-N-LG_Rocky) was excluded in this study which was only used for the comparison between two different dissociation protocols: Collagenases+Dispase and Rocky.
Replication	Biological replicates from multiple patients were included in our data. Replication within single cell RNA sequencing is defined as recurrent results in all patients. In this study we have 19 patients, hence 19 replicates of our results.
Randomization	The patients were recruited randomly in this study. 19 treatment-naïve patients were diagnosed with prostate adenocarcinoma and underwent radical prostatectomy. We separate patients into different groups based on tumor grades score (low-grade: Gleason 6 and 7; high-grade:Gleason 8-10). Benign prostate gland tissue adjacent to the tumor was collected as adj-normal. In vivo experiments were done on C57Bl6 mice ordered from Jackson Laboratory and were of the same age and gender (males). For treatment, mice were randomized when tumor volume reached average of 300mm3.

Blinding	Investigators were blinded at data collection and experimental group was checked after conducting the experiment.

Reporting for specific materials, systems and methods

We require information from authors about some types of materials, experimental systems and methods used in many studies. Here, indicate whether each material, system or method listed is relevant to your study. If you are not sure if a list item applies to your research, read the appropriate section before selecting a response.

Materials & experimental systems

n/a	Involved in the study
☐	☒ Antibodies
☐	☒ Eukaryotic cell lines
☒	☐ Palaeontology and archaeology
☐	☒ Animals and other organisms
☒	☐ Clinical data
☒	☐ Dual use research of concern

Methods

n/a	Involved in the study
☒	☐ ChIP-seq
☐	☒ Flow cytometry
☒	☐ MRI-based neuroimaging

Antibodies

Antibodies used	Human sort for scRNAseq: anti-human CD235-PE (Biolegend, Catalogue# 306604, Clone: HIR2, lot# B260964)<br><br>For multiplex Immunohistochemistry: CD3 (Rabbit polyclonal, Dako), CD8 (C8/144B, Mouse monoclonal, Dako), PD-1 (Cell signaling, Catalogue# 43248S, Clone: EH33, Mouse monoclonal), FOXP3 (Cell signaling, Catalogue# 74816, Clone: D2W8E, Rabbit monoclonal), CD68 (PG-M1, Mouse monoclonal, Dako), CD163 (Leica Biosystem, Catalogue# CD163-L-CE, Clone: 10D6, Mouse monoclonal). Secondary antibody conjugated to horse radish peroxidase (HRP), and an opal fluorophore (Opal 690, Opal 570, Opal 540, Opal 620, Opal 650 and Opal 520, Akoya Biosciences).<br><br>The antibodies used for flow sorting has been added as a supplementary Data 7. For CCL20 blocking antibody (R&D, cat#MAB7601, and Rat IgG isotype control from R&D of cat#MAB005, anti-PD1 from BioXcell of cat#BE0146 and its IgG2a isotype control from BioXcell of cat#BE0089).
Validation	Validation of primary antibodies as determined by manufacturer. The antibodies has been verified to be used on human samples and quality tested to be used on Flow Cytometry.

Eukaryotic cell lines

Policy information about cell lines and Sex and Gender in Research

Cell line source(s)	RM1 cells (male), ATCC#CRL-3310
Authentication	We didnot do authentication for RM1 cells.
Mycoplasma contamination	The cells were regularly checked for mycoplasma and tested negative.
Commonly misidentified lines (See ICLAC register)	No commonly misidentified line was involved in this study.

Animals and other research organisms

Policy information about studies involving animals; ARRIVE guidelines recommended for reporting animal research, and Sex and Gender in Research

Laboratory animals	All animals were C57Bl6 mice obtained from Jackson Laboratories of age 6-8 weeks old at the time of experiment start. The housing conditions for the mice are as the following:<br>Lighting - rodent rooms is 12:12, light:dark (7 am – 7 pm)<br>Bedding type - Hardwood Sanichip; with Carefresh nesting material<br>Rodent housing - either Allentown Inc (rectangular caging, 160 cages per IVC rack; which uses blower at 60 ACH) or Animal Care Systems (pie-shaped caging, 100 cages per round rack using house exhaust)<br>Room Temp = 68 – 73*F<br>Relative Humidity = 30-70%
Wild animals	No wild animals were used in the study.
Reporting on sex	All animals used in our study were males.

Field-collected samples	No field collected samples were used in the study.
Ethics oversight	All animal studies were done in accordance Institutional Animal Care and Use Committee of Massachusetts General Hospital guidelines.

Note that full information on the approval of the study protocol must also be provided in the manuscript.

Flow Cytometry

Plots

Confirm that:

☒ The axis labels state the marker and fluorochrome used (e.g. CD4-FITC).

☒ The axis scales are clearly visible. Include numbers along axes only for bottom left plot of group (a 'group' is an analysis of identical markers).

☒ All plots are contour plots with outliers or pseudocolor plots.

☒ A numerical value for number of cells or percentage (with statistics) is provided.

Methodology

Sample preparation	Dissociation of tissues into single cells: All samples were collected in Media 199 supplemented with 2% (v/v) FBS. Single cell suspensions of the tumors were obtained by cutting the tumor in to small pieces (1mm3) followed by enzymatic dissociation for 45 minutes at 37°C with shaking at 120 rpm using Collagenase I, Collagenase II, Collagenase III, Collagenase IV (all at a concentration of 1mg/ml) and Dispase (2mg/ml) in the presence of RNase inhibitors (RNasin (Promega), RNase OUT (Invitrogen)), and DNase I (ThermoFisher). Erythrocytes were subsequently removed by ACK Lysing buffer (Quality Biological) and cells resuspended in Media 199 supplemented with 2% (v/v) FBS for further analysis.<br>FACS sorting: Single cells from tumor samples were surface stained with anti-CD235-PE (Biolegend) for 30 min at 4°C. Cells were washed twice with 2% FBS-PBS (v/v) followed by DAPI staining (1 ug/ml). Flow sorting for live-nonerythroid cells (DAPI-neg/CD235-neg) was performed on a BD FACS Aria III instrument equipped with a 100um nozzle (BD Biosciences, San Jose, CA). All flow cytometry data were analyzed using FlowJo software (FlowJo 10.8.1, Treestar, San Carlos, CA).
Instrument	BD FACS Aria III instrument (BD Biosciences, San Jose, CA).
Software	FlowJo software (FlowJo 10.8.1, Treestar, San Carlos, CA).
Cell population abundance	For all samples that were cell sorted for single-cell profiling by 10X, live cells that were CD235 negative (e.g. erythroid cells) were 80-90% of total cells.
Gating strategy	Cells were selected first based on FSC-A and SCC-A followed by singlet selection for both FSC and SSC. Then live cells were selected based on DAPI staining (DAPI negative) followed by selection of CD235 negative cells which were sorted for 10x experiments (DAPIneg CD235neg).

☒ Tick this box to confirm that a figure exemplifying the gating strategy is provided in the Supplementary Information.
